# Biodegradation of flonicamid by *Ensifer adhaerens* CGMCC 6315 and enzymatic characterization of the nitrile hydratases involved

**DOI:** 10.1186/s12934-021-01620-4

**Published:** 2021-07-13

**Authors:** Yun-Xiu Zhao, Li Wang, Ke-Xin Chen, Neng-Dang Jiang, Shi-Lei Sun, Feng Ge, Yi-Jun Dai

**Affiliations:** 1 Jiangsu Key Laboratory for Microbes and Functional Genomics, Jiangsu Engineering and Technology Research Center for Industrialization of Microbial Resources, College of Life Science, Nanjing Normal University, Nanjing, 210023 People’s Republic of China; 2 The Key Laboratory of Biotechnology for Medicinal Plants of Jiangsu Province and School of Life Science, Jiangsu Normal University, Xuzhou, 221116 People’s Republic of China; 3 Nanjing Institute of Environmental Sciences, Ministry of Environmental Protection, Nanjing, 210042 People’s Republic of China

**Keywords:** Biodegradation, Cell immobilization, *Ensifer adhaerens* CGMCC 6315, Enzymatic degradation, Flonicamid

## Abstract

**Background:**

Flonicamid (*N*-cyanomethyl-4-trifluoromethylnicotinamide, FLO) is a new type of pyridinamide insecticide that regulates insect growth. Because of its wide application in agricultural production and high solubility in water, it poses potential risks to aquatic environments and food chain.

**Results:**

In the present study, *Ensifer adhaerens* CGMCC 6315 was shown to efficiently transform FLO into *N*-(4-trifluoromethylnicotinoyl) glycinamide (TFNG-AM) via a hydration pathway mediated by two nitrile hydratases, PnhA and CnhA. In pure culture, resting cells of *E. adhaerens* CGMCC 6315 degraded 92% of 0.87 mmol/L FLO within 24 h at 30 °C (half-life 7.4 h). Both free and immobilized (by gel beads, using calcium alginate as a carrier) *E. adhaerens* CGMCC 6315 cells effectively degraded FLO in surface water. PnhA has, to our knowledge, the highest reported degradation activity toward FLO, *V*_max_ = 88.7 U/mg (*K*_m_ = 2.96 mmol/L). Addition of copper ions could increase the enzyme activity of CnhA toward FLO by 4.2-fold. Structural homology modeling indicated that residue *β*-Glu56 may be important for the observed significant difference in enzyme activity between PnhA and CnhA.

**Conclusions:**

Application of *E. adhaerens* may be a good strategy for bioremediation of FLO in surface water. This work furthers our understanding of the enzymatic mechanisms of biodegradation of nitrile-containing insecticides and provides effective transformation strategies for microbial remediation of FLO contamination.

**Supplementary Information:**

The online version contains supplementary material available at 10.1186/s12934-021-01620-4.

## Highlights

1. *E. adhaerens* efficiently degrades the insecticide FLO via a hydration pathway.
2. Both free and immobilized cells effectively degrade FLO in surface water.
3. *E. adhaerens* nitrile hydratases CnhA and PnhA both hydrolyze FLO to TFNG-AM.
4. PnhA has, to our knowledge, the highest reported degradation activity toward FLO.
5. The key residue (*β*-Glu56) may cause a significant difference in two NHase activities.

**Supplementary Information:**

The online version contains supplementary material available at 10.1186/s12934-021-01620-4.

## Introduction

Flonicamid (*N*-cyanomethyl-4-trifluoromethylnicotinamide, FLO) is a novel systemic insecticide with selective activity that exhibits very good efficacy in pest control [1–3]. It is widely applied for foliar treatment of cabbages, tea trees, dwarf berry crops, and fruits [4–6]. FLO and its metabolites *N*-(4-trifluoromethylnicotinoyl)glycinamide (TFNG-AM), 5-trifluoromethylnicotinic acid, and 4-(trifluoromethyl)nicotinol glycine were detected in orange groves in field studies [6, 7]. FLO residues have also been detected in human serum and urine samples, and several watersheds around the Great Lakes Basin in the United States [8, 9]. High doses of FLO caused DNA degradation and severe genomic damage in mice [10]. Because of their high solubility in water, these compounds can remain in the edible parts of food and enter the food chain [10–12]. The presence of these long-lasting compounds in the environment poses potential risks to human health.

Microbial catabolism of pesticides is one of the most important and effective methods for pesticide decomposition [13, 14]. Previously, we have shown that microbes may be one of the major factors affecting FLO degradation in soil [15]. *Microvirga flocculans* CGMCC 1.16731, *Aminobacter* sp. CGMCC 1.17253, and *Ensifer meliloti* CGMCC 7333 can each convert FLO to TFNG-AM in pure culture via hydration [16–18]; *Alcaligenes faecalis* CGMCC 17553 can transform FLO via hydrolysis and hydration pathways. Some reports indicate that the degradation of FLO is rapid in soil, with a maximum DT_90_ (the time required for 90% dissipation of the initial concentration) of 1.5–8.7 days, which is far below the trigger value of 100 days [19]. Per kilogram dry weight of soil, the LC_50_ value of FLO was > 1000 mg, which indicates that FLO poses a low risk to earthworms and soil microorganisms [20]. However, FLO has higher persistence in water and the total water–sediment system, with DT_50_ values of 30–37 and 36–44 d, respectively (https://www.ohp.com/Labels_MSDS/PDF/pradia_sds.pdf). Microbial remediation of FLO in surface water environments has not yet been reported. The degradation behavior of FLO and its mechanisms are increasing concerns.

The microbial degradation of nitriles proceeds via two enzymatic pathways: (i) the nitrile hydratase/amidase pathway, and (ii) the nitrilase pathway [21]. Nitrile hydratase (NHase, EC 4.2.1.84) is one of the key enzymes for nitrile metabolism in microorganisms. It catalyzes the hydration of nitriles to the corresponding amides, and shows great application potential in the degradation of toxic nitrile compounds [22–24]. NHases are heteromultimers composed of *α*- and *β*-subunits with either a non-heme iron (Fe-NHase) or a non-corrin cobalt ion (Co-NHase) in the active site [25]. Gene cloning and overexpression analysis identified two NHases in *E. adhaerens* CGMCC 6315, located on the chromosome (CnhA) and a plasmid (PnhA), respectively, that were responsible for conversion of the neonicotinoid acetamiprid [26]. However, these NHases have not been fully biochemically and structurally characterized.

In this study, we applied free and immobilized *E. adhaerens* CGMCC 6315 cells for remediation of FLO in surface water. We also characterized the NHases CnhA and PnhA from this bacterium; they degrade FLO, and PnhA shows high activity. These results enhance our understanding of FLO degradation and develop a good agent for FLO bioremediation.

## Materials and methods

### Chemicals and media

FLO (C_9_H_6_F_3_N_3_O, CAS Registry No. 158062-67-0, 95% purity) was purchased from Hubei Zhengxingyuan Fine Chemical Co. (Wuhan, China). TFNG-AM (C_9_H_8_F_3_N_3_O_2_, CAS Registry No. 158062-96-5, 99% purity) was prepared as described previously [15, 16]. High-performance liquid chromatography (HPLC)-grade acetonitrile was supplied by Merck (Darmstadt, Germany). All other reagents were of analytical grade and were supplied by Sangon Biotech (Shanghai, China).

Luria–Bertani (LB) medium (10 g/L NaCl, 10 g/L tryptone, and 5 g/L yeast extract, pH 7.2) was used for cultivation of all *Escherichia* (*Es*.) *coli* strains. The nutrient concentration of LB was diluted to give 1/15 LB, which was used for cultivation of *E. adhaerens* CGMCC 6315 [26].

### Strains and plasmids

*E. adhaerens* CGMCC 6315 and *Es. coli* Rosetta (DE3) harboring the *E. adhaerens* CGMCC 6315 NHase-encoding genes (*cnhA* and *pnhA*) are stored in our laboratory [26, 27]. *Es. coli* Rosetta (DE3) pLysS (Novagen, USA) served as the host strain for protein expression experiments, and pET28a (+) (Novagen, Germany) was used as the expression vector [26]. The accession numbers of the *α*-subunit gene, *β*-subunit gene, and activator gene (the genes encoding PnhA) in the GenBank database are MH998515, MH998516, and MH998517, respectively. The accession numbers of the *α*-subunit gene, *β*-subunit gene, and activator gene (the genes encoding CnhA) in GenBank are MH998512, MH998513, and MH998514, respectively.

### Kinetics of FLO degradation by resting cells of *E. adhaerens* CGMCC 6315

*E. adhaerens* CGMCC 6315 were inoculated into a 100-mL flask containing 20 mL LB medium and incubated in a rotary shaker (220 rpm) at 30 °C. After incubation for 16 h, 1 mL of this seed culture was inoculated into a 500-mL flask containing 150 mL 1/15LB medium supplemented with CoCl_2_ (final concentration of 0.1 mmol/L) and incubated for 72 h. Cells were harvested by centrifugation at 7000×g for 8 min. The cell sediments obtained were washed twice with 50 mmol/L sodium phosphate buffer (pH 7.5). The cell density was adjusted to OD_600_ = 5 and then resuspended in 5 mL of the same buffer containing 0.87 mmol/L FLO. The reaction system was placed on a rotary shaker (220 rpm) at 30 °C. Samples were taken at intervals, centrifuged at 12,000×*g* for 10 min to remove cells, and the supernatant was collected, filtered, and diluted to a volume appropriate for analysis of the FLO and metabolites by HPLC.

### Biodegradation of FLO in surface water by free and immobilized cells

Surface water samples were collected from Jiuxiang Lake, Nanjing, China, and then filtered through sterilized 0.22-µm Millipore filter membranes. FLO in surface water samples is additionally added. Water samples (10 mL each) containing 0.21 mmol/L FLO were poured into 100-mL flasks and then resting cells of *E. adhaerens* CGMCC 6315 were added to a final concentration of 1 × 10^9^ colony-forming units (CFU)/mL. Surface water with no added bacterial cells was used as a control. These flasks were incubated at 30 °C, 120 rpm. At 24-h intervals, the supernatant was collected and prepared for HPLC analysis as described above [28].

For examination of the FLO-degradation ability of immobilized cells, 4 mL of seed culture broth were transferred into 1-L flasks containing 350 mL of 1/15 LB medium supplemented with CoCl_2_ (final concentration of 0.1 mmol/L) and incubated for 3 d (30 °C, 200 rpm). The cells were harvested, washed twice with 50 mmol/L sodium phosphate buffer (pH 7.5), and finally suspended in sterilized deionized water containing 4% sodium alginate. The mixture was thoroughly stirred and dropped into CaCl_2_ (2% w/v) solution through a 10-mL injector. Gel beads with a diameter of about 2–3 mm were formed and calcified for 24 h [29, 30]. Beads giving a final bacterial concentration of 1.25 × 10^9^ CFU/mL were transferred into 500-mL flasks holding 100-mL of surface water containing 0.21 mmol/L FLO. These flasks were then incubated at 30 °C, 150 rpm. At 2-d intervals, samples were collected and prepared for HPLC analysis as described above.

### HPLC and liquid chromatography-mass spectrometry (LC–MS) analyses

An Agilent 1260 HPLC system was used for quantitative analysis of FLO and its metabolites. The HPLC system used an Agilent reverse phase HC-C18 column (4.6 × 250 mm) equipped with a reverse phase C18 pre-column (4.6 × 20 mm). The mobile phase was deionized water containing 0.01% acetic acid and acetonitrile (water: acetonitrile, 70:30 v:v). Elution was conducted at a flow rate of 1 mL/min and monitored at 265 nm using an Agilent G1314A UV detector. For LC–MS analysis, an Agilent 1290 infinity liquid chromatograph with a G1315B diode array detector and an Agilent 6460 triple quadrupole LC–MS system equipped with an electrospray ion source (Agilent Technologies) were used. LC–MS analysis used the same mobile phase as HPLC, but the flow rate was 0.6 mL/min.

### Biodegradation of FLO by resting cells of *Es. coli* Rosetta (DE3) overexpressing NHase from *E. adhaerens* CGMCC 6315

We examined the FLO-degradation ability by resting cells of *Es. coli* pET28a-*pnhA* (expressing *E. adhaerens* CGMCC 6315 NHase PnhA) and *Es. coli* pET28a-*cnhA* (expressing *E. adhaerens* CGMCC 6315 NHase CnhA). *Es. coli*-pET28a cells were used as a control. Initially, bacteria were inoculated into a 100-mL flask containing 30 mL LB medium and incubated in a rotary shaker (37 °C, 220 rpm). After incubation for 12 h, 1 mL of this seed culture was inoculated into a 500-mL flask containing 150 mL LB medium and incubated for ~ 2.5 h (until OD_600_ reached 0.5). Then isopropyl *β*-d-1-thiogalactopyranoside was added to a final concentration of 0.2 mmol/L. After incubation for 6 h, the cells were harvested by centrifugation at 9000×*g* for 8 min. The cell sediments were washed with 50 mmol/L sodium phosphate buffer (pH 7.5). The cell density was adjusted to OD_600_ = 5 in 5 mL of the same buffer containing 0.87 mmol/L FLO. After transformation for 2 h, the samples were centrifuged at 10,000×*g* for 8 min to remove the residual cells and the supernatant was collected, filtered, and diluted to a volume appropriate for analysis of the substrate and metabolites by HPLC.

### Enzyme purification and biochemical characterization

Details of the overexpression and purification of the two recombinant NHases were as reported in our previous studies [26, 31]. The *E. adhaerens* CGMCC 6315 NHases were respectively overexpressed in *Es. coli* Rosetta (DE3) with an *N*-terminal 6 × His-tag and purified by affinity chromatography according to the instructions of the chromatography resin manufacturer (Novagen Inc., Madison, WI, USA). Sodium dodecyl sulfate–polyacrylamide gel electrophoresis (SDS-PAGE) was used to assess protein expression, and gels were stained using Coomassie Brilliant Blue R-250. The concentrations of the separating gel and focusing gel were 12.5% and 5% (w/v), respectively.

The optimal reaction pH and temperature for degradation of FLO were determined by measuring NHase activity in different buffers at pH 4–9 (citrate buffer pH 4.0–6.0, sodium phosphate buffer pH 6.0–8.0, Tris–HCl buffer pH 8.0–9.0) and at 20–70 °C, respectively. To test the pH stability of NHase activity, the purified enzyme was preincubated at 4 °C for 12 h in FLO-containing buffers with different pH values and the residual activity was determined. Thermal stability was determined by preincubating the enzyme at 20–70 °C for 2 h, and the residual activity was measured. The effects of metal ions on NHase activity were measured after adding EDTA, CaCl_2_, CuSO_4_, FeCl_3_, MnCl_2_, ZnCl_2_, NaCl, CoCl_2_, or MgCl_2_ to the reaction mixture at a final concentration of 1 mmol/L. The effects of organic solvents on NHase activity were measured by individually adding dimethyl sulfoxide (DMSO), ethanol, methanol, dichloromethane, ethyl acetate, acetone, cyclohexane, or 1-butanol (at a volume ratio of 2%) to the reaction mixture. Substrate specificities of the two NHases were tested by separately adding 2 mmol/L FLO, acetamiprid (ACE), thiacloprid (THI), indole-3-acetonitrile (IAN), 3-cyanopyridine (3-CP), dichlobenil, bromoxynil, or fipronil to the reaction mixture and then assaying by HPLC. For kinetic analysis, reactions with a range of FLO concentrations were performed at 37 °C. Kinetic constants were calculated using nonlinear regression analysis (Michaelis–Menten) in Origin 8.6 software [16, 32, 33].

NHase activity was determined using HPLC analysis. One unit (U) of NHase activity was defined as the amount of enzyme that catalyzed the formation of 1 µmol of TFNG-AM in 1 min. The reaction with total volume of 1 mL was conducted for 10 min at 37 °C and quenched by the addition of 500 µL acetonitrile. Then, the samples were centrifuged at 10,000×*g* for 5 min and the supernatants were analyzed by HPLC.

### Half-life determination

Half-life values for the degradation of FLO were determined by plotting ln(*I/I*_0_) against time [based on the equation ln(*I/I*_0_) =  − *kt*, where *I*_0_ and *I* represent the initial and residual concentrations, respectively]. The half-life (*t*_1/2_) was calculated as *t*_1/2_ = (ln2)/*k*, where *k* is the apparent elimination constant. The first-order equation provided a satisfactory fit for the data (r > 0.9) [34].

### Structural homology modeling of NHases

Structural homology models of the NHases from *E. adhaerens* CGMCC 6315 were constructed using Phyre2 (http://www.sbg.bio.ic.ac.uk/phyre2/html/) and the SWISS-MODEL website (https://swissmodel.expasy.org/interactive). The crystal structures of NHase subunits from *Pseudonocardia thermophila* and *Pseudomonas putida* (PDB accession codes 4ob1.1.A, 3qyg.1.B, 3qxe.1.A and 3qz9.1.B) [35, 36] were used as templates for the *α*-subunit of PnhA, the *β*-subunit of PnhA, the *α*-subunit of CnhA, and the *β*-subunit of CnhA, respectively. Global model quality estimation (GMQE) and quantitative model energy analysis (QMEAN) were used to assess the quality of the constructed NHase structures [16].

## Results and discussion

### The kinetics of FLO degradation by *E. adhaerens* CGMCC 6315 and metabolite identification

**Fig. 1:**
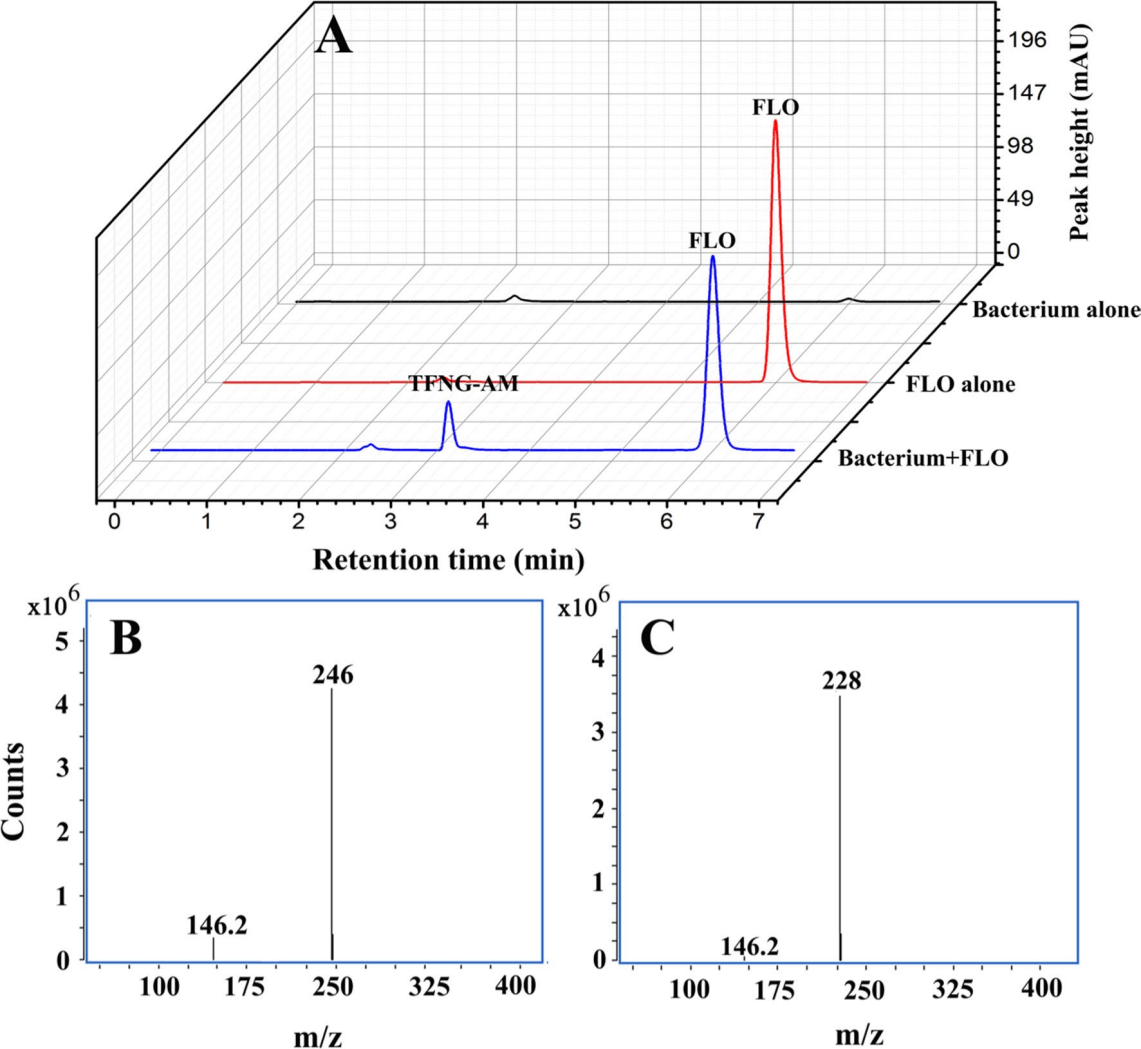
HPLC and LC − MS analysis. **A** HPLC analysis of the degradation of FLO by *E. adhaerens* CGMCC 6315. **B** Negative ion mass spectrum of TFNG-AM. **C** Negative ion mass spectrum of FLO

*E. adhaerens* CGMCC 6315 metabolized FLO to one apparent polar metabolite with retention time 3.25 min by HPLC analysis (Fig. 1A), which corresponds to the retention time of TFNG-AM in LC–MS. This peak did not appear in the substrate or bacterium controls (Fig. 1A). In mass spectra in negative ion mode (Fig. 1B and C), the metabolite and substrate had peaks at *m/z* 246 and 228, respectively, corresponding to [M−H]^−^ ions. A common fragment ion was also observed at *m/z* 146.2, which is consistent with C_6_H_5_NF_3,_ already reported in a previous study [4]. Some reports have shown that the molecular weights of TFNG-AM and FLO are 247 and 229 [15, 16]. Therefore, the metabolite of FLO was identified as TFNG-AM. These results indicate that *E. adhaerens* CGMCC 6315 can metabolize FLO to TFNG-AM via hydration.

**Fig. 2:**
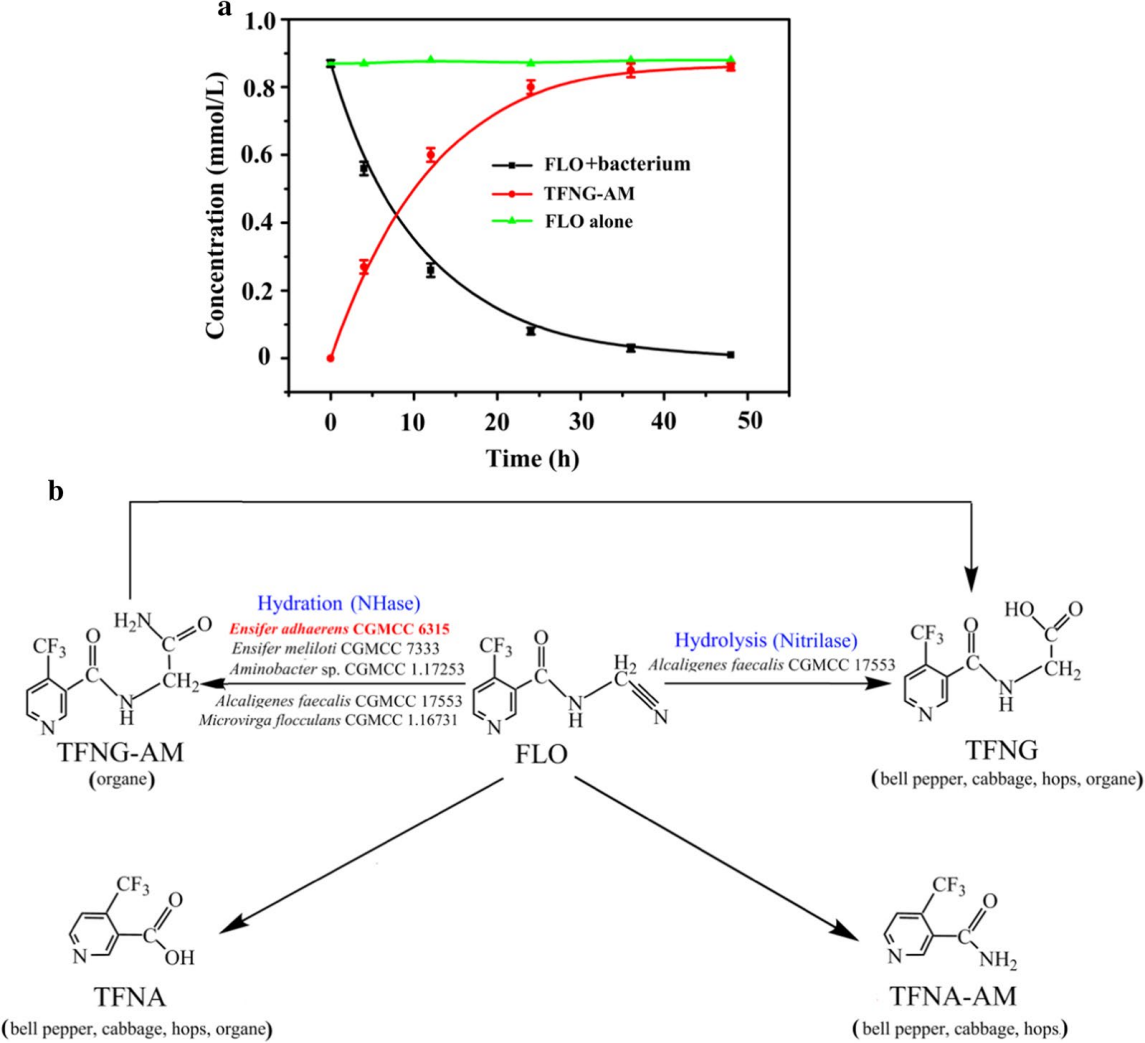
**A** FLO biodegradation by *E. adhaerens* CGMCC 6315 in pure culture, and **B** proposed metabolic pathways

Resting cells of *E. adhaerens* CGMCC 6315 degraded FLO from an initial concentration of 0.87 mmol/L to 0.07 mmol/L in 24 h (92% FLO degradation) in Fig. 2A. Meanwhile, 0.79 mmol/L TFNG-AM was formed (molar conversion 98.8%). Thus, TFNG-AM is the main product of FLO hydrolysis. The half-life of FLO in the presence of *E. adhaerens* CGMCC 6315 was only 7.4 h, significantly shorter than that in FLO degradation by *A. faecalis* CGMCC 17553 (15 h), *E. meliloti* CGMCC 7333 (60 h), and *Aminobacter* sp. CGMCC 1.17253 (178.8 h) [15, 17, 18]. Therefore, *E. adhaerens* CGMCC 6315 may be more advantageous for microbial restoration of FLO-contaminated environments. Metabolic pathways of FLO degradation in various microbes and the FLO metabolites in common fruit and vegetable crops are shown in Fig. 2B.

### Biodegradation of FLO in surface water by free and immobilized *E. adhaerens* CGMCC 6315 cells

**Fig. 3:**
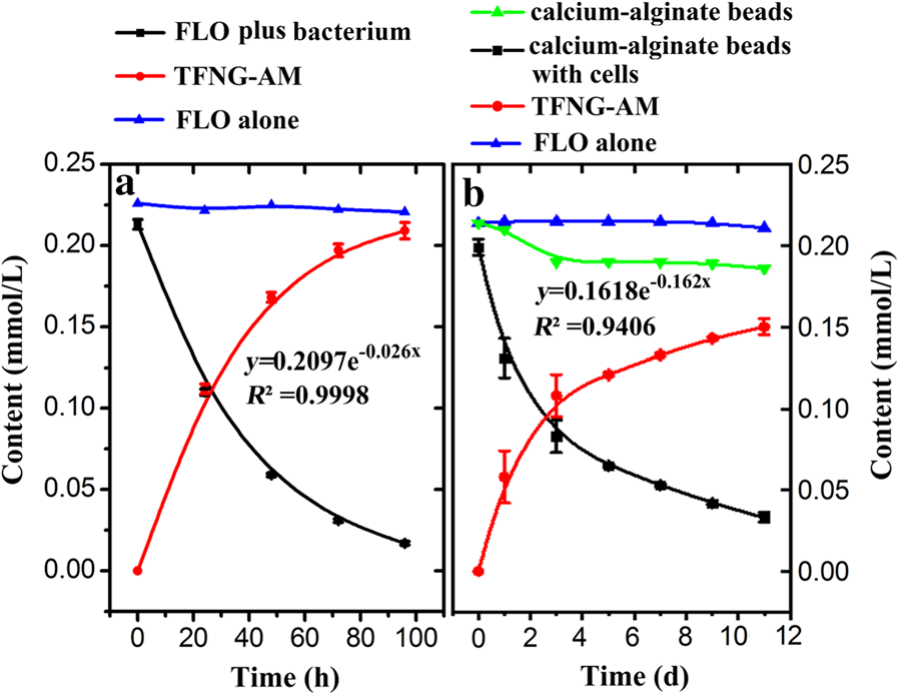
Time course of FLO degradation by *E. adhaerens* CGMCC 6315 in surface water. **A** Free cells; **B** cells immobilized by gel beads using calcium alginate as a carrier

FLO is highly soluble in water and can remain as residue in the edible parts of crops that enter the food chain [12]. *E. adhaerens* CGMCC 6315 was inoculated into surface water to examine its ability to degrade FLO. After incubation for 4 d, the FLO content was reduced from the initial value of 0.21 mmol/L to 0.01 mmol/L (95.2% degradation) in Fig. 3A. The control without bacterial inoculation had no activity toward FLO.

* E. adhaerens* CGMCC 6315 cells immobilized by gel beads using calcium alginate as a carrier were also prepared for evaluation of FLO degradation ability in surface water. The control beads adsorbed some FLO over the first 2 d; 11.1% of the initial FLO was adsorbed (Fig. 3B). The immobilized cells degraded 78.9% of the FLO after 11 d of incubation. These results indicate that *E. adhaerens* CGMCC 6315 has the potential to degrade FLO in surface water. When using free cells to degrade toxic substances in wastewater treatment, there are problems such as difficulty in handling, decrease in cell density, and reduction of adaptation and infiltration rates. However, cell immobilization technology can provide protection against harsh environmental conditions and prolong the survival of microorganisms [37]. The immobilization of microbial cells has attracted increasing attention in the field of wastewater treatment [30]. Compared with conventional wastewater treatment systems, immobilized cell systems have high potential to degrade toxic chemicals. In addition, the cost of biological treatment is much lower than that of physical and chemical methods [38].

We previously isolated an effective thiacloprid-degrading strain, *M. flocculans* CGMCC 1.16731, which showed weak FLO degradation ability in surface water, but immobilized cells barely degraded FLO (data not shown) [16]. As a nitrogen-fixing bacterium, application of *M. flocculans* is usually limited to microbe–plant combined remediation. *E. adhaerens*, as a nitrogen-fixing and plant growth-promoting rhizobacterium, is a common inhabitant of soil and water environments, and shows great potential to decompose complex organic pollutants [39]. Zhou et al. [27] reported that *E. adhaerens* breaks down the pesticide thiamethoxam and produces secondary metabolites that are beneficial to plant growth and germination. In the present study, *E. adhaerens* CGMCC 6315 is shown to be capable of removing FLO from surface water.

### Bioinformatic analysis of the NHases of *E. adhaerens* CGMCC 6315, expression of the NHases in *Es. coli* Rosetta (DE3), and degradation of FLO

**Fig. 4:**
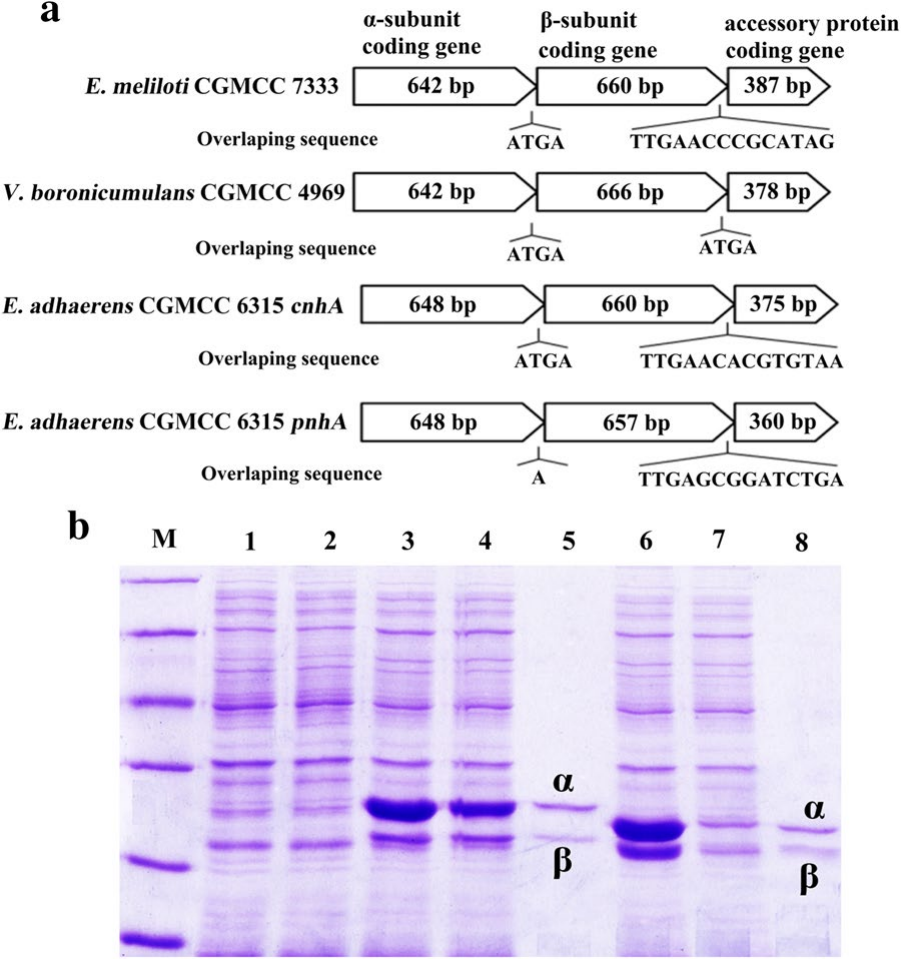
Expression of *E. adhaerens* CGMCC 6315 PnhA and CnhA in *Escherichia coli*. (A) Comparison of the NHase gene clusters in *E. meliloti* CGMCC 7333, *V. boronicumulans* CGMCC 4969, and *E. adhaerens* CGMCC 6315. **B** SDS-PAGE analysis of expression of *E. adhaerens* CGMCC 6315 NHases in *Es. coli*. Lane M, protein molecular weight markers (116, 66.2, 45, 35, 25 and 18.4 kDa); lanes 1 and 2, the total and soluble protein from *Es. coli* control cells; lanes 3 and 4, the total and soluble protein from *Es. coli* cells expressing *E. adhaerens* CGMCC 6315 CnhA; lane 5, purified CnhA; lanes 6 and 7, the total and soluble protein from *Es. coli* cells expressing *E. adhaerens* CGMCC 6315 PnhA; lane 8, purified PnhA

*Es. coli* Rosetta (DE3) overexpressing *E. adhaerens* CGMCC 6315 NHase genes were constructed in our previous study [26]. *E. adhaerens* CGMCC 6315 contains genes encoding two nitrile hydratases, one (CnhA) encoded on the chromosome and the other (PnhA) on a plasmid. Their NHase gene clusters composition in *E. adhaerens* CGMCC 6315 is ⟨α-subunit⟩, ⟨β-subunit⟩, and ⟨activator protein⟩. *cnhA* has gene structure *α*-subunit (648 bp), *β*-subunit (660 bp), and activator protein (375 bp). There is a four-base (ATGA) overlapping sequence between the *α*-subunit and *β*-subunit genes, and there is an overlapping sequence of 14 bases (TTGAACACGTGTAA) between the *β*-subunit gene and the activator gene, which is similar to our previous report for *E. meliloti* CGMCC 7333 nitrile hydratase (Fig. 4A). *pnhA* has gene structure *α*-subunit (648 bp), *β*-subunit (657 bp), and activator protein (360 bp). The overlapping sequence between the *α*-subunit and *β*-subunit genes is simply the base “A”, which differs from the four bases in *cnhA*, *E. meliloti* CGMCC 7333 NHase, *Variovorax boronicumulans* CGMCC 4969 NHase [28, 33], and all other overlapping sequences previously reported for nitrile hydratases.

Phylogenetic analysis based on the amino acid sequences of NHase *α*-subunits indicated that CnhA clustered in a branch with the NHase of other FLO degrading bacteria, *E. meliloti* CGMCC 7333 and *Aminobacter* sp*.* CGMCC 1.17253 [17, 33], with 82.33 and 74.42% protein sequence similarity respectively. Comparing *E. adhaerens* CGMCC 6315 PnhA and CnhA with the enzymes from *E. meliloti* CGMCC 7333, the protein similarities were 51.63% and 82.33%, respectively. *E. adhaerens* CGMCC 6315 PnhA and CnhA clustered in different branches, indicating evolutionary divergence (Additional file 1: Figure S1).

SDS-PAGE analysis suggested that the solubility of CnhA was good (Fig. 4B, lane 4). In contrast, PnhA was less soluble and more inclusion bodies were observed (Fig. 4B, lane 7). Lanes 5 and 8 represent the purified CnhA and PnhA, respectively. Activator protein bands were not observed. We speculate that the reason may be a low expression level of the activator protein, which is similar to the previously reported *mf*NHase from another FLO-degrading bacterium, *M. flocculans* CGMCC 1.16731 [32]. Resting cells of *Es. coli* pET28a-*pnhA* and *Es. coli* pET28a-*cnhA* respectively exhibited FLO degradation activity, while control cells (*Es. coli*-pET28a) had no activity toward FLO. The results indicated that PnhA and CnhA each degrade FLO to TFNG-AM via hydration.

### Enzymatic characterization of *E. adhaerens* CGMCC 6315 CnhA

**Fig. 5:**
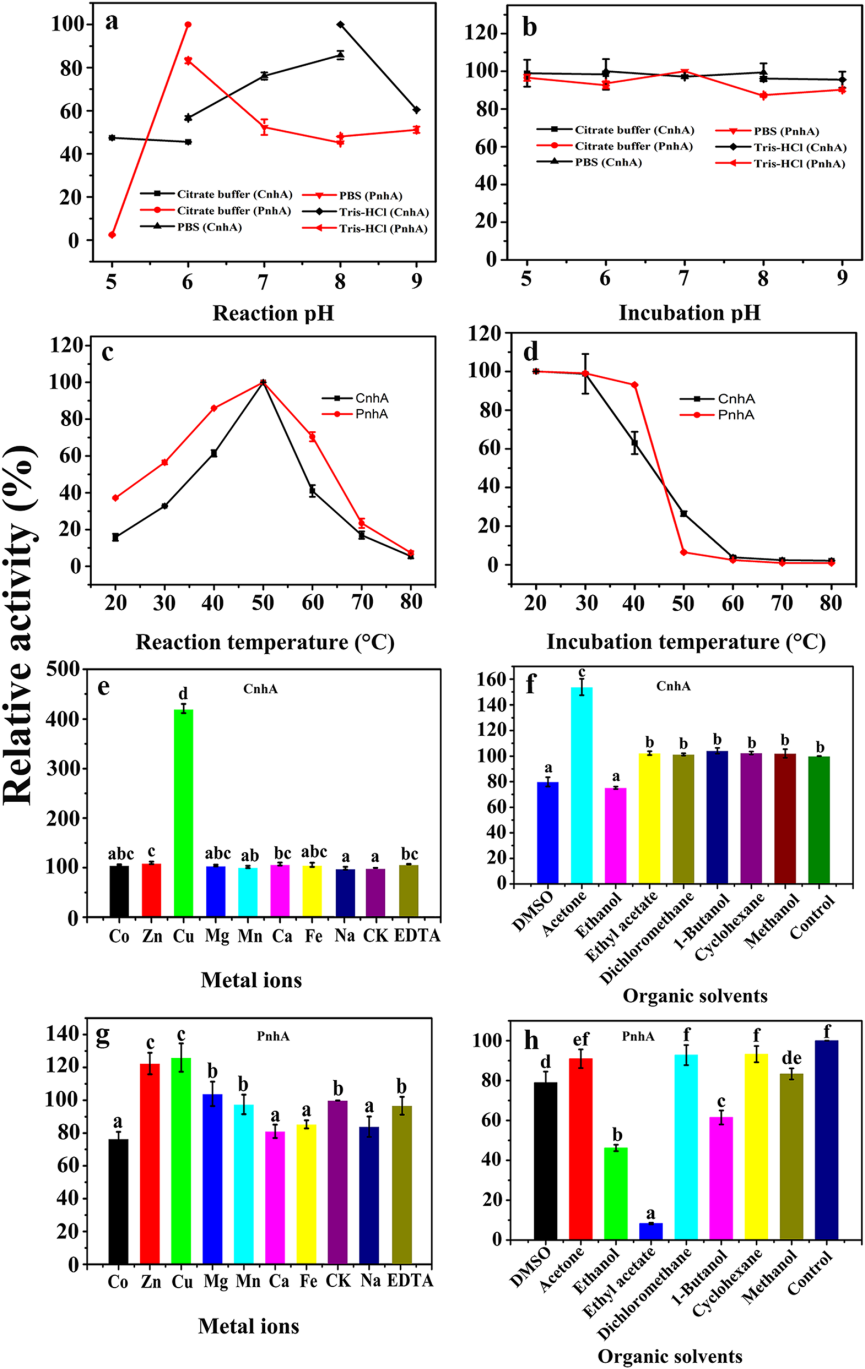
Enzymatic characterization of *E. adhaerens* CGMCC 6315 CnhA and PnhA. **A** Effects of pH on the activity of CnhA and PnhA. **B** Effects of pH on the stability of CnhA and PnhA. **C** Effects of temperature on the activity of CnhA and PnhA. **D** Effects of temperature on the stability of CnhA and PnhA. **E** Effects of metal ions and **F** organic solvents on the activity of CnhA. **G** Effects of metal ions and **H** organic solvents on the activity of PnhA. Enzyme assays used FLO as the substrate. Average values and standard deviations were calculated from triplicate samples from three parallel cultures (*n* = 9). Different letters (a–f) above the columns indicate significant differences at *p* ≤ 0.05 according to the Duncan test

The optimal pH for FLO hydration by CnhA was 8.0 (Fig. 5A), and the enzyme activity reached the highest. At pH 5.0, the enzyme activity was only 47.6% of the maximum activity, while it dramatically inhibited FLO hydration by 52.4%. Preincubation of CnhA for 12 h at different pH 5–9 had only a slight effect on the NHase activity toward FLO; the residual activity remained > 95.6% (Fig. 5B). CnhA exhibited its maximum FLO degradation activity at 50 °C. When the reaction temperature was increased to 60 °C, the enzyme activity decreased markedly (Fig. 5C). When the pure enzyme was preincubated for 2 h at > 40 °C, the activity dramatically declined. After preincubation for 2 h at 60 °C, CnhA had almost no activity (Fig. 5D). When the preincubation temperature exceeded 40 °C, *Aminobacter* sp. CGMCC 1.17253 NHase activity also decreased dramatically, like that of CnhA [17]. PnhF from *M. flocculans* CGMCC 1.6731 was preincubated for 2 h at 20–60 °C and the residual activity remained at about 60% [16].

Addition of many types of metal ion slightly promoted CnhA activity in hydration of FLO. However, strikingly, addition of Cu^2+^ ions increased the activity by 4.2-fold compared with the control treatment (no added metal ions) (Fig. 5E). This promotion of activity by Cu^2+^ ions was not found for the NHases from other FLO-degrading bacteria (*A. faecalis* CGMCC 17553, *Aminobacter* sp. CGMCC 1.17253, *E. meliloti* CGMCC 7333 and *V. boronicumulans* CGMCC 4969) [15, 17, 18, 28]. We speculate that Cu^2+^ ions may promote enzyme folding, thereby forming a larger amount of NHase with the correct conformation and hence increasing the enzyme activity [40]. Among the tested organic solvents, DMSO and ethanol inhibited the activity of CnhA in hydration of FLO by 20.1% and 24.75%, respectively. Acetone increased the activity in hydration of FLO by 1.54-fold (Fig. 5F).

Analysis of kinetic parameters showed that the process of FLO degradation by CnhA accorded with Michaelis–Menten kinetics (Additional file 1: Figure S2). The Michaelis constant was 5.07 mmol/L, and *V*_max_ was 9.55 U/mg. The Michaelis constant and *V*_max_ of PnhF from *M. flocculans* CGMCC 1.16731 involved in the formation of TFNG-AM from FLO were 32.9 mmol/L and 5.9 U/mg (Table 1), which indicated that CnhA had a higher affinity for FLO than PnhF.

**Table 1:** Biochemical properties of purified enzymes from FLO-degrading bacteria

Name	Protein	Microorganism	*V*_max (U/mg)_	*K*_m (mM)_	Optimum reaction		References
					pH	Temperature	
NHase	PnhA	*E. adhaerens* CGMCC 6315	88.7	2.96	6	50 °C	This study
NHase	CnhA	*E. adhaerens* CGMCC 6315	9.55	5.07	8	50 °C	This study
NHase	PnhF	*M. flocculans* CGMCC 1.16731	5.9	32.9	5	50 °C	Zhao et al. [16]
NHase	ANHase	*Aminobacter* sp. CGMCC 1.17253	14.98 × 10^–3^	21.03	7	40 °C	Yang et al. [17]
Nitrilase	NitA	*A. faecalis* CGMCC 17553	0.58	0.59	7	40 °C	Yang et al. [15]
Nitrilase	NitD	*A. faecalis* CGMCC 17553	0.18 × 10^–3^	145.87	8	40 °C	Yang et al. [15]

Enzyme characterization indicated that *E. adhaerens* CnhA has notable tolerance to a range of pH, metal ions, and organic solvents, and may have application potential in repairing environmental pollution. Oves et al. [41] reported that *E. adhaerens* OS3 can not only biosorb 95% of the Ni and 74% of the Pb under laboratory condition; it can also produce and secrete plant-promoting biomass. Zhou and Sun [26, 27] reported that *E. adhaerens* CGMCC 6315 could degrade the neonicotinoids thiamethoxam and acetamiprid, and promote the germination rate of soybeans under salt stress. *E. adhaerens* has already been widely used in agricultural production, but its application in the remediation of pesticide pollutants is still relatively rare.

### Enzymatic characterization of *E. adhaerens* CGMCC 6315 PnhA

The optimal pH for FLO hydration by PnhA was 6.0 (Fig. 5A), and the enzyme activity reached the highest. At pH 5.0, the PnhA activity only retained 2.43% of the maximum activity. Preincubation of PnhA for 12 h at pH 5–9 buffer had a slight effect on the NHase activity toward FLO; the residual activity was  > 86.98% (Fig. 5B). PnhA showed its maximum activity toward FLO at 50 °C; the enzyme showed only 23.38% of the maximum activity at 70 °C (Fig. 5C). When the enzyme was preincubated for 2 h at > 40 °C, its activity dramatically declined; indeed, it had almost no activity after preincubation at ≥ 50 °C (Fig. 5D).

On addition of metal ions, all the tested ions except Mg^2+^, Zn^2+^ and Cu^2+^ inhibited the degradation activity of PnhA toward FLO. Addition of Zn^2+^ and Cu^2+^ ions increased the activity by 1.2- and 1.26-fold, respectively compared with the control treatment (no added metal ions) (Fig. 5G). Furthermore, promotion by Cu^2+^ ions was also found for CnhA. However, obviously, the promoting effect of Cu^2+^ ions on CnhA is much higher than that of PnhA. Compared with CnhA, all organic solvents tested inhibited the activity of PnhA toward FLO. In particular, compared with the control treatment, ethyl acetate and ethanol inhibited the activity by 91.68% and 53.85%, respectively (Fig. 5H). These results indicated that PnhA is more sensitive to organic solvents.

Analysis of kinetic parameters showed that the process of FLO degradation by PnhA accorded with Michaelis–Menten kinetics (Additional file 1: Figure S2). The Michaelis constant was 2.96 mmol/L, and *V*_max_ was 88.7 U/mg. The *V*_max_ values of NitA, NitD, PnhF and *Aminobacter* sp. CGMCC 1.17253 NHase involved in the formation of TFNG-AM from FLO were 0.58 U/mg, 0.18 mU/mg, 5.9 U/mg, and 14.98 mU/mg, respectively (Table 1), much lower than the *V*_max_ of PnhA. As far as we know, PnhA has the highest degradation activity toward FLO yet reported.

Both CnhA and PnhA could degrade FLO, THI, 3-CP, IAN, and ACE. The activity of PnhA toward THI, 3-CP and ACE was much higher than that of CnhA, but its ability to transform IAN was much lower than that of CnhA (Table 2). We speculate that this is because of the structure of IAN, which means that it binds more easily to the active-site pocket of CnhA than PnhA. Both NHases had no degradation activity toward fipronil, dichlobenil or bromoxynil (Table 2). Our results indicate that CnhA and PnhA both exhibit strict substrate specificity.

**Table 2:** Substrate specificities of purified CnhA and PnhA

Substrate	CAS Registry No	Specific activity ± Standard deviation (U/mg)	
		CnhA	PnhA
FLO	158062-67-0	4.30 ± 0.12b	34.12 ± 1.32c
ACE	135,410-20-7	0.85 ± 0.01a	21.34 ± 0.17b
THI	111988-49-9	7.65 ± 0.04c	58.09 ± 1.38d
3-CP	100-54-9	158.25 ± 3.86e	625.62 ± 4.24e
IAN	771-51-7	21.28 ± 0.79d	9.15 ± 0.39a
Fipronil	120068-37-3	ND	ND
Dichlobenil	1194-65-6	ND	ND
Bromoxynil	1689-84-5	ND	ND

NHase activity was assayed with 2 mmol/L substrate. Data indicate the means of three replicates. Values are the mean ± SD. Different letters adjacent to the values indicate statistically significant differences (Duncan test, p ≤ 0.05)

ND: no activity detected

### Homology modelling of PnhA and CnhA

The amino acid sequence similarities between the templates and the *E. adhaerens* CGMCC 6315 protein subunits of interest were 39.18%, 36.87%, 62.87%, and 44.91%, respectively (Additional file 1: Figure S3). The GMQE values were 0.15, 0.75, 0.80 and 0.78, and the QMEAN values were − 3.41, − 3.47, − 0.24 and − 2.10, respectively. The GMQE value is a number between 0 and 1, where higher numbers indicate higher reliability. A QMEAN score near 0 indicates that the model structure is in good agreement with experimental structures of similar size; a score of − 4.0 or below indicates that the quality of the model is low [42].

**Fig. 6:**
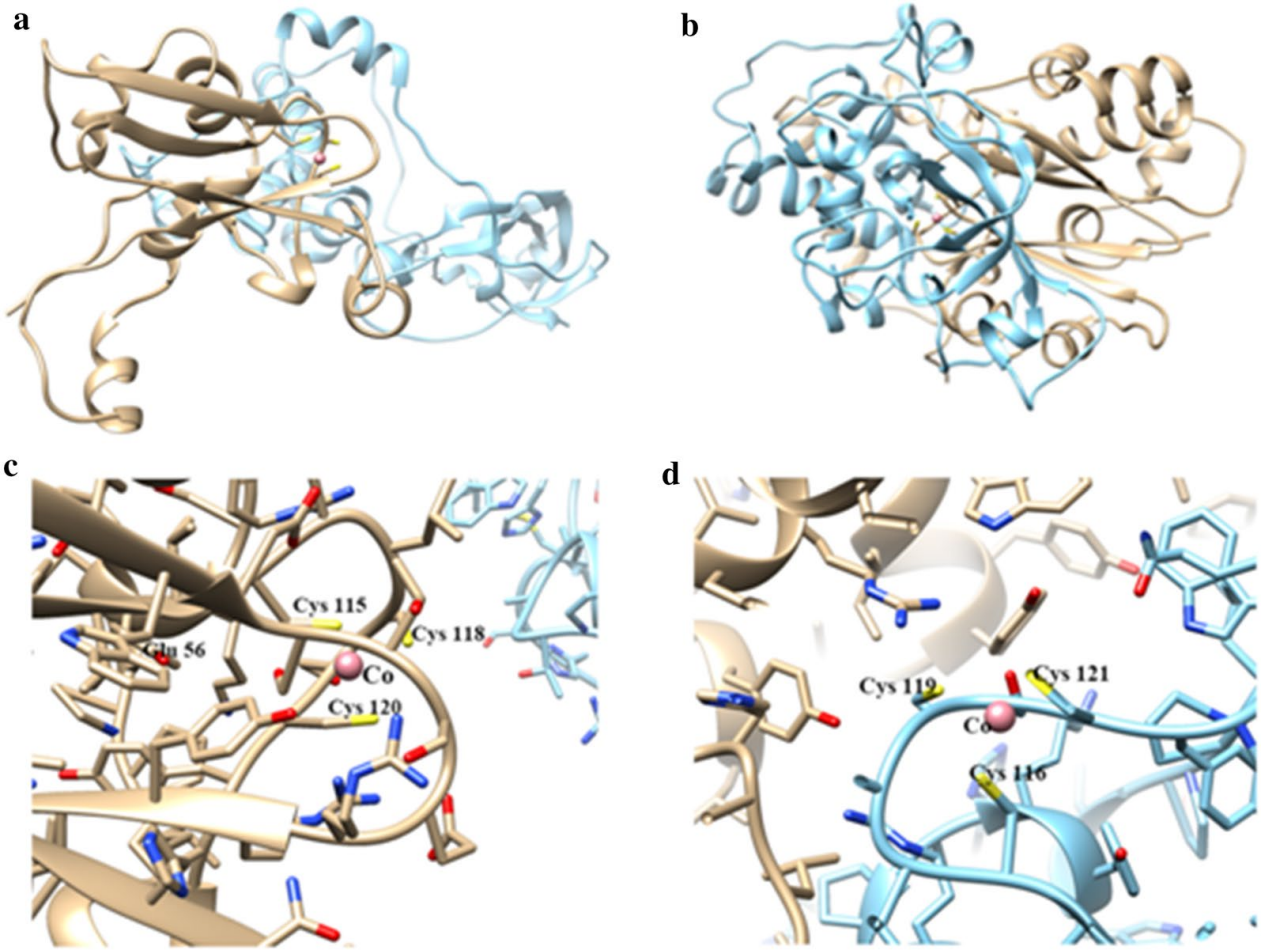
Homology models of *E. adhaerens* CGMCC 6315 PnhA and CnhA. **A** PnhA, **B** CnhA. Predicted key active site residues of **C** PnhA and **D** CnhA are marked, and the cobalt ion is shown as a light pink sphere

The three-dimensional structural models of PnhA and CnhA are shown in Fig. 6A and B. The metal coordination sphere in the *α*-subunit of PnhA involves residues Cys115–Thr116–Leu117–Cys118–Ser119–Cys120 (Fig. 6C). Cys118 and Cys120, which coordinate the cobalt ion, were post-translationally oxidized to sulfinic and sulfenic acid, respectively. In the α-subunit of CnhA, residues Cys116–Thr117–Leu118–Cys119–Ser120–Cys121 were in the coordination sphere of the cobalt ion; Cys119 and Cys121 of CnhA play the same role as Cys118 and Cys120 of PnhA (Fig. 6D) [43–45]. The post-translational oxidation of these residues has also been observed in the NHases from *M. flocculans* CGMCC 1.16731 and *Streptomyces canus* CGMCC 13662 [16, 46].

The second-shell residues *β*-Glu56 and *β*-His147 (far from the active site) play important roles in the catalytic activity of *P. putida* NHase [36, 46]. The key amino residue Glu-56 was present in the *β*-subunit of *E. adhaerens* CGMCC 6315 PnhA (Fig. 6C). However, the corresponding amino residue was not found in CnhA. Both PnhA and CnhA can transform FLO, but the specific activity of PnhA was much higher than that of CnhA. We speculate that CnhA may lack other key residues, resulting in the large difference in enzyme activity.

## Conclusions

In this study, we found that *E. adhaerens* CGMCC 6315 efficiently degrades the insecticide FLO, and showed that two NHases from this bacterium, PnhA and CnhA, mediate the hydrolysis of FLO to metabolite TFNG-AM. Both free and immobilized *E. adhaerens* CGMCC 6315 cells were found to effectively degrade FLO in surface water. PnhA has the highest degradation activity toward FLO of any NHase yet reported. CnhA is more tolerant to a wide range of pH, heavy metal ions, and organic solvents. These findings could help to generate effective strategies for microbial remediation of FLO contamination.

## Supplementary Information

**Additional file 1: Figure S1.** Phylogenetic analysis of NHases; **Figure S2.** Kinetic parameters of FLO degradation reactions catalyzed by *E. adhaerens* CGMCC 6315 PnhA and CnhA; **Figure S3.** Alignment of sequences of *E. adhaerens* CGMCC 6315 NHases PnhA and CnhA with sequences of NHases from *Pseudonocardia thermophila* and *Pseudomonas putida* that were used as templates for homology modelling.

## Data Availability

All data generated or analyzed during this study are included in this published article and its additional files.

## References

[1] Wang S, Jin F, Cao X, Shao Y, Wang J, She Y, Qi Y, Zhang C, Li H, Jin M, Wang J, Shao H, Zheng L (2018). Residue behaviors and risk assessment of flonicamid and its metabolites in the cabbage field ecosystem. Ecotoxicol Environ Saf.

[2] Korr V (2007). Teppeki (R) - a new insecticide for tillage (wheat and potatoes). Gesunde Pflanzen.

[3] Chawla S, Gor HN, Patel HK, Parmar KD, Patel AR, Shukla V, Ilyas M, Parsai SK, Meena RS, Shah PG (2018). Validation, residue analysis, and risk assessment of fipronil and flonicamid in cotton (*Gossypium* sp.) samples and soil. Environ Sci Pollut Res.

[4] López-Ruiz R, Romero-González R, Vidal JLM, Frenich AG (2016). Determination of flonicamid and its metabolites in bell pepper using ultra-high-performance liquid chromatography coupled to high-resolution mass spectrometry (Orbitrap). Food Addit Contam Part A.

[5] Abdel-Ghany MF, Hussein LA, El Azab NF (2017). Multiresidue analysis of five neonicotinoid insecticides and their primary metabolite in cucumbers and soil using high-performance liquid chromatography with diode-array detection. J AOAC Int.

[6] Sabry AKH, Salem LM, Ali NI, Ahmed SSE (2018). Genotoxic effect of flonicamid and etofenprox on mice. Biosci Res.

[7] Hengel MJ, Miller M (2007). Analysis of flonicamid and its metabolites in dried hops by liquid chromatography-tandem mass spectrometry. J Agric Food Chem.

[8] López-Ruiz R, Ruiz-Muelle AB, Romero-González R, Fernández I, Martínez Vidal JL, Frenich AG (2017). The metabolic pathway of flonicamid in oranges using an orthogonal approach based on high-resolution mass spectrometry and nuclear magnetic resonance. Anal Methods.

[9] Metcalfe CD, Helm P, Paterson G, Kaltenecker G, Murray C, Nowierski M, Sultana T (2019). Pesticides related to land use in watersheds of the Great Lakes basin. Sci Total Environ.

[10] Yamamuro T, Ohta H, Aoyama M, Watanabe D (2014). Simultaneous determination of neonicotinoid insecticides in human serum and urine using diatomaceous earth-assisted extraction and liquid chromatography-tandem mass spectrometry. J Chromatogr B.

[11] López A, Yusà V, Millet M, Coscollà C (2016). Retrospective screening of pesticide metabolites in ambient air using liquid chromatography coupled to high-resolution mass spectrometry. Talanta.

[12] Masiá A, Suarez-Varela MM, Llopis-Gonzalez A, Picó Y (2016). Determination of pesticides and veterinary drug residues in food by liquid chromatography-mass spectrometry: a review. Anal Chim Acta.

[13] Fan X, Song F (2014). Bioremediation of atrazine: recent advances and promises. J Soils Sediments.

[14] Xiong H, Dong S, Zhang J, Zhou D, Rittmann BE (2018). Roles of an easily biodegradable co-substrate in enhancing tetracycline treatment in an intimately coupled photocatalytic-biological reactor. Water Res.

[15] Yang WL, Guo LL, Dai ZL, Qin RC, Zhao YX, Dai YJ (2019). Biodegradation of the insecticide flonicamid by *Alcaligenes faecalis* CGMCC 17553 via hydrolysis and hydration pathways mediated by nitrilase. J Agric Food Chem.

[16] Zhao YX, Yang WL, Guo L, Jiang HY, Cheng X, Dai YJ (2020). Bioinformatics of a novel nitrile hydratase gene cluster of the N2-fixing bacterium *Microvirga flocculans* CGMCC 1.16731 and characterization of the enzyme. J Agric Food Chem.

[17] Yang WL, Dai ZL, Cheng X, Fan ZX, Jiang HY, Dai YJ (2020). Biotransformation of insecticide flonicamid by *Aminobacter* sp. CGMCC 1.17253 via nitrile hydratase catalysed hydration pathway. J Appl Microbiol..

[18] Yang WL, Fan ZX, Jiang HY, Zhao YX, Guo L, Dai YJ (2021). Biotransformation of flonicamid and sulfoxaflor by multifunctional bacterium *Ensifer meliloti* CGMCC 7333. J Environ Sci Health Part B.

[19] European Food Safety Authority (2010). Conclusion on the peer review of the pesticide risk assessment of the active substance flonicamid. EFSA J.

[20] Australian Pesticides and Veterinary Medicines Authority: Public release summary on the evaluation of the new active flonicamid in the product Mainman 500 WG insecticide. APVMA Product Number P66373, 2014, Kingston, Australia. https://apvma.gov.au/sites/default/files/publication/13721-prs-flonicamid.pdf.

[21] Sakashita T, Hashimoto Y, Oinuma KI, Kobayashi M (2008). Transcriptional regulation of the nitrile hydratase gene cluster in *Pseudomonas chlororaphis* B23. J Bacteriol.

[22] Supreetha K, Rao SN, Srividya D, Anil HS, Kiran S (2019). Advances in cloning, structural and bioremediation aspects of nitrile hydratases. Mol Biol Rep.

[23] Yamada H, Kobayashi M (1996). Nitrile hydratase and its application to industrial production of acrylamide. Biosci Biotechnol Biochem.

[24] Kobayashi M, Shimizu S (1998). Metalloenzyme nitrile hydratase: structure, regulation, and application to biotechnology. Nat Biotechnol.

[25] Hopmann KH, Guo JD, Himo F (2007). Theoretical investigation of the first-shell mechanism of nitrile hydratase. Inorg Chem.

[26] Sun SL, Fan ZX, Zhao YX, Guo L, Dai YJ (2019). A novel nutrient deprivation-induced neonicotinoid insecticide acetamiprid degradation by *Ensifer adhaerens* CGMCC 6315. J Agric Food Chem.

[27] Zhou GC, Wang Y, Zhai S, Liu ZH, Dai YJ (2013). Biodegradation of the neonicotinoid insecticide thiamethoxam by the nitrogen-fixing and plant-growth-promoting rhizobacterium *Ensifer adhaerens* strain TMX-23. Appl Microbiol Biotechnol.

[28] Sun SL, Yang WL, Guo JJ, Zhou YN, Rui X, Ge F, Dai YJ (2017). Biodegradation of the neonicotinoid insecticide acetamiprid in surface water by the bacterium *Variovorax boronicumulans* CGMCC 4969 and its enzymatic mechanism. RSC Adv.

[29] Dai ZL, Yang WL, Fan ZX, Guo L, Liu ZH, Dai YJ (2021). Actinomycetes *R**hodococcus ruber* CGMCC 17550 degrades neonicotinoid insecticide nitenpyram via a novel hydroxylation pathway and remediates nitenpyram in surface water. Chemosphere.

[30] Lin HY, Chen ZL, Megharaj M, Naidu R (2013). Biodegradation of TNT using *Bacillus mycoides* immobilized in PVA-sodium alginate-kaolin. Appl Clay Sci.

[31] Guo LL, Yang WL, Cheng X, Fan ZX, Chen XM, Ge F, Dai YJ (2021). Degradation of neonicotinoid insecticide acetamiprid by two different nitrile hydratases of *Pseudaminobacter salicylatoxidans* CGMCC 117248. Int Biodeterior Biodegrad.

[32] Zhao YX, Jiang HY, Cheng X, Zhu YX, Fan ZX, Dai ZL, Guo L, Liu ZH, Dai YJ (2019). Neonicotinoid thiacloprid transformation by the N2-fixing bacterium *Microvirga flocculans* CGMCC 1.16731 and toxicity of the amide metabolite. Int Biodeterior Biodegrad.

[33] Sun SL, Lu TQ, Yang WL, Guo JJ, Rui X, Mao SY, Zhou LY, Dai YJ (2016). Characterization of a versatile nitrile hydratase of the neonicotinoid thiacloprid-degrading bacterium *Ensifer meliloti* CGMCC 7333. RSC Adv.

[34] Wang G, Chen X, Yue W, Zhang H, Li F, Xiong M (2013). Microbial degradation of acetamiprid by *Ochrobactrum* sp. D-12 isolated from contaminated soil. PLoS ONE.

[35] Martinez S, Wu R, Sanishvili R, Liu D, Holz R (2014). The active site sulfenic acid ligand in nitrile hydratases can function as a nucleophile. J Am Chem Soc.

[36] Brodkin HR, Novak WRP, Milne AC, D’Aquino JA, Karabacak NM, Goldberg IG, Agar JN, Payne MS, Petsko GA, Ondrechen MJ, Ringe D (2011). Evidence of the participation of remote residues in the catalytic activity of Co-type nitrile hydratase from *Pseudomonas putida*. Biochemistry.

[37] Conde-Avila V, Ortega-Martínez LD, Loera O, El Kassis EG, Dávila JG, Valenzuela CM, Armendáriz BP (2020). Pesticides degradation by immobilised microorganisms. Int J Environ Anal Chem..

[38] Wang JL, Quan XC, Han LP, Qian Y, Hegemann W (2002). Microbial degradation of quinoline by immobilized cells of *Burkholderia pickettii*. Water Res.

[39] Harada N, Takagi K, Baba K, Fujii K, Iwasaki A (2010). Biodegradation of diphenylarsinic acid to arsenic acid by novel soil bacteria isolated from contaminated soil. Biodegradation.

[40] Kobayashi M, Shimizu S (1999). Cobalt proteins. Eur J Biochem.

[41] Oves M, Khan MS, Qari HA (2017). *Ensifer adhaerens* for heavy metal bioaccumulation, biosorption, and phosphate solubilization under metal stress condition. J Taiwan Instit Chem Engineers.

[42] Benkert P, Künzli M, Schwede T (2009). QMEAN server for protein model quality estimation. Nucleic Acids Res.

[43] Stępkowski T, Banasiewicz J, Granada CE, Andrews M, Passaglia LMP (2018). Phylogeny and phylogeography of rhizobial symbionts nodulating legumes of the tribe genisteae. Genes.

[44] Phugare SS, Jadhav JP, Testing R (2015). Biodegradation of acetamiprid by isolated bacterial strain *Rhodococcus* sp. BCH2 and toxicological analysis of its metabolites in silkworm (*Bombax mori*). Clean Soil Air Water.

[45] Hashimoto K, Suzuki H, Taniguchi K, Noguchi T, Yohda M, Odaka M (2008). Catalytic mechanism of nitrile hydratase proposed by time-resolved X-ray crystallography using a novel substrate, *tert*-butylisonitrile. J Biol Chem.

[46] Guo L, Fang WW, Guo LL, Yao CF, Zhao YX, Ge F, Dai YJ (2019). Biodegradation of the neonicotinoid insecticide acetamiprid by actinomycetes *Streptomyces canus* CGMCC 13662 and characterization of the novel nitrile hydratase involved. J Agric Food Chem.

