# Supplementary material for: Biodegradation of flonicamid by Ensifer adhaerens CGMCC 6315 and enzymatic characterization of the nitrile hydratases involved
Source: Microb Cell Fact. 2021 Jul 13;20:133. doi: 10.1186/s12934-021-01620-4 (PMC8278588; doi:10.1186/s12934-021-01620-4)
Supplement: Supplementary file 1 — Additional file 1: Figure S1. Phylogenetic analysis of NHases; Figure S2. Kinetic parameters of FLO degradation reactions catalyzed by E. adhaerens CGMCC 6315 PnhA and CnhA; Figure S3. Alignment of sequences of E. adhaerens CGMCC 6315 NHases PnhA and CnhA with sequences of NHases from Pseudonocardia thermophila and Pseudomonas putida that were used as templates for homology modelling. [file 12934_2021_1620_MOESM1_ESM.docx]

**Supporting Information**

**Biodegradation of flonicamid by *Ensifer adhaerens* CGMCC 6315 and enzymatic characterization of the nitrile hydratases involved**

Yun-Xiu Zhao ^a^ • Li Wang ^a^ • Ke-Xin Chen ^a^ • Neng-Dang Jiang ^a^ • Shi-Lei Sun ^b^ • Feng Ge ** ^c^ • Yi-Jun Dai* ^a^

^a^Jiangsu Key Laboratory for Microbes and Functional Genomics, Jiangsu Engineering and Technology Research Center for Industrialization of Microbial Resources, College of Life Science, Nanjing Normal University, Nanjing 210023, People’s Republic of China

^b^The Key Laboratory of Biotechnology for Medicinal Plants of Jiangsu Province and School of Life Science, Jiangsu Normal University, Xuzhou 221116, People’s Republic of China

^c^Nanjing Institute of Environmental Sciences, Ministry of Environmental Protection, Nanjing 210042, People’s Republic of China

*****Corresponding author. [daiyijun@njnu.edu.cn](mailto:daiyijun@njnu.edu.cn) (Yi-Jun Dai) (86-25-85891731).

******Corresponding author. [gefeng@nies.org](mailto:gefeng@nies.org) (Feng Ge).

Authors’ email addresses: [18852099577@163.com](mailto:18852099577@163.com) (Yun-Xiu Zhao), [1692589637@qq.com](mailto:1692589637@qq.com) (Li Wang), [858574310@qq.com](mailto:858574310@qq.com) (Ke-Xin Chen), [741616646@qq.com](mailto:741616646@qq.com) (Neng-Dang Jiang), [1159290841@qq.com](mailto:1159290841@qq.com) (Shi-Lei Sun).

**Fig S1. Phylogenetic analysis of NHases**

**
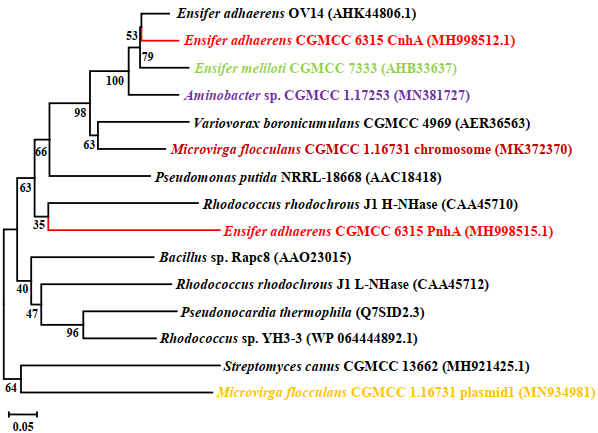
**

The scale bar indicates 0.05 substitutions per nucleotide position.

**Fig S2. Kinetic parameters of FLO degradation reactions catalyzed by *E. adhaerens* CGMCC 6315 PnhA and CnhA**


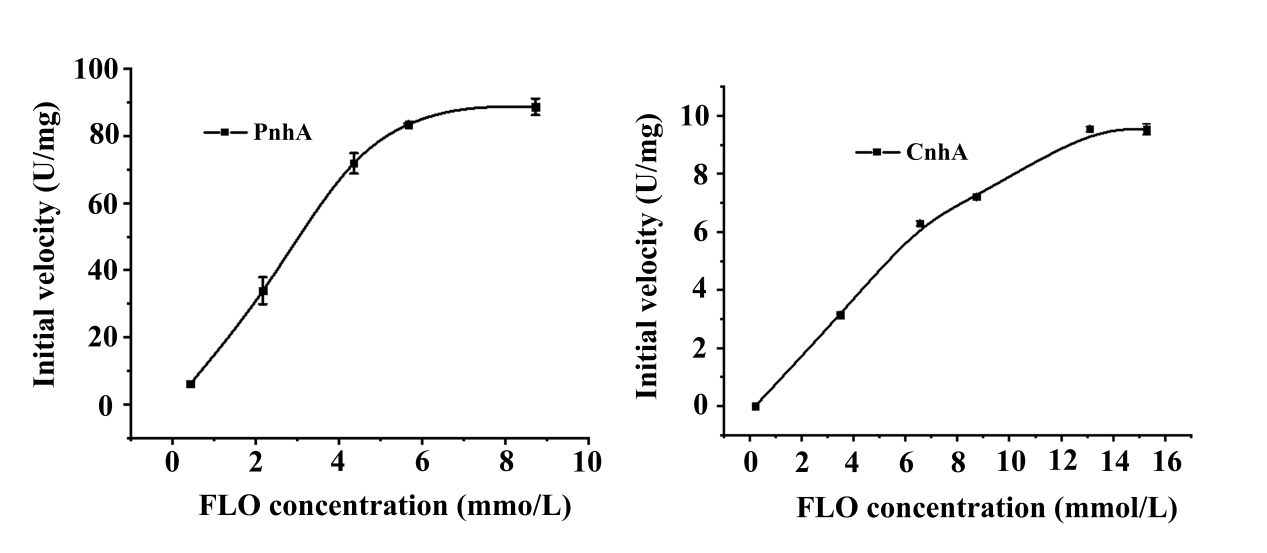


**Fig S3. Alignment of sequences of *E. adhaerens* CGMCC 6315 NHases PnhA and CnhA with sequences of NHases from *Pseudonocardia thermophila* and *Pseudomonas putida* that were used as templates for homology modelling**

**
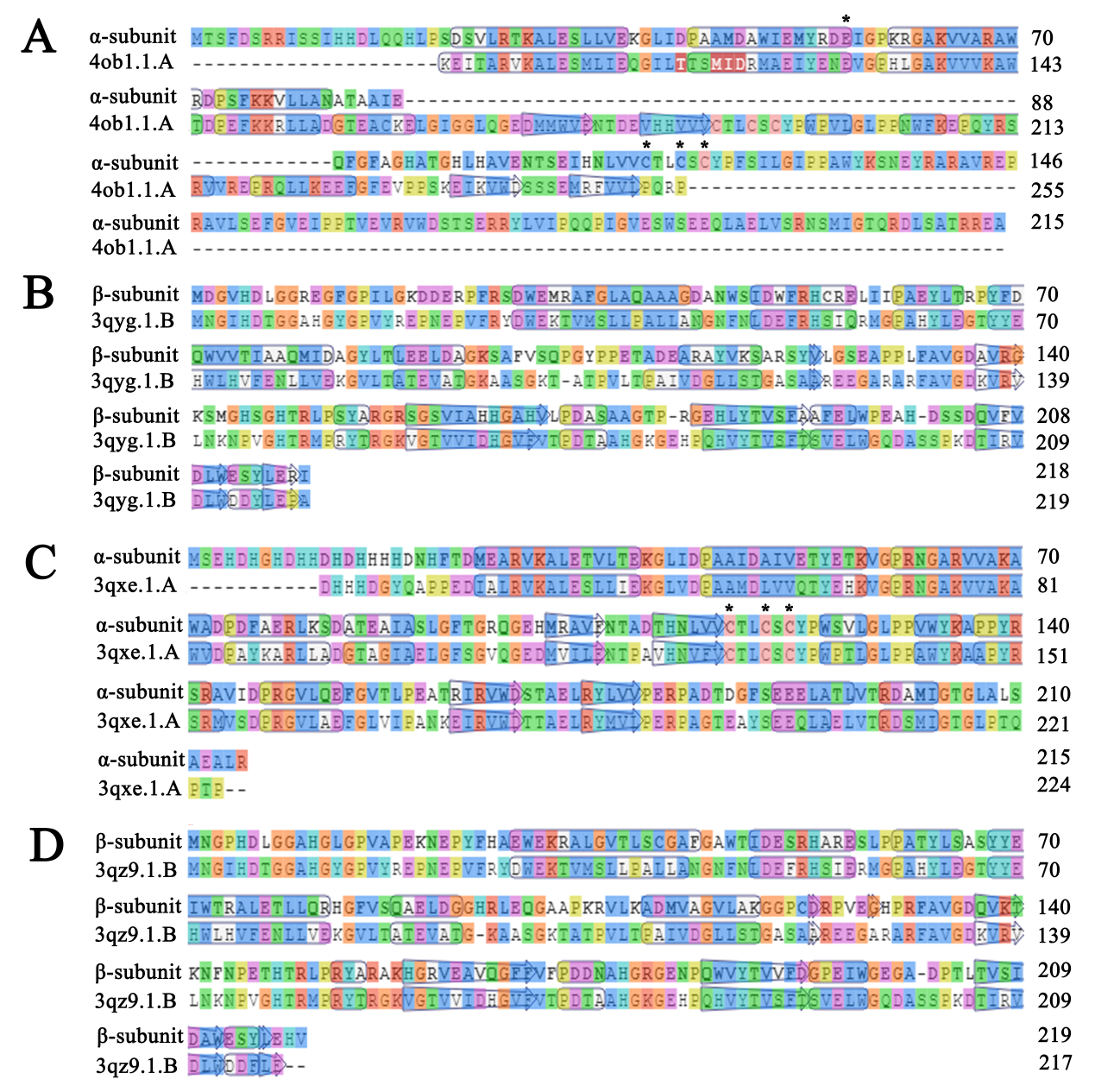
**

*: The important conserved amino acid site.
